# Non‐alcoholic fatty liver disease and the risk of fibrosis in Italian primary care services: GPS‐NAFLD Study

**DOI:** 10.1111/liv.15443

**Published:** 2022-10-18

**Authors:** Luca Miele, Ignazio Grattagliano, Francesco Lapi, Marianxhela Dajko, Antonio De Magistris, Antonio Liguori, Nicoletta De Matthaeis, Alessandro Rossi, Antonio Gasbarrini, Claudio Cricelli, Antonio Grieco

**Affiliations:** ^1^ DiSMeC – Department of Scienze Mediche e Chirurgiche Fondazione Policlinico Gemelli IRCCS Rome Italy; ^2^ Department of Medicina e Chirurgia Traslazionale Università Cattolica Del Sacro Cuore Rome Italy; ^3^ SIMG Italian College of General Practitioners and Primary Care Florence Italy; ^4^ Health Search Italian College of General Practitioners and Primary Care Florence Italy

## Abstract

**Background and aims:**

The prevalence of non‐alcoholic fatty liver disease (NAFLD) is increasing globally. This study aimed to determine the prevalence of NAFLD and the probability of liver fibrosis in Italian primary care services.

**Methods:**

We carried out a population‐based and nested case–control study including all individuals aged 18 years and above registered at Italian primary care services. Data were collected from the general practitioners' network from 2010 to 2017. NAFLD cases were identified via the ICD‐9‐CM and Hepatic Steatosis Index score > 36 and were matched each up to 10 controls. Other causes of liver diseases were excluded. The risk of fibrosis was assessed using the FIB‐4 and NAFLD fibrosis scores (NFS).

**Results:**

NAFLD was present in 9% of the primary care population with high regional variability. Among NAFLD subjects: 25% had diabetes, 10% had chronic kidney disease, 11% had cardiovascular disease and 28% were obese. Furthermore, 30% had at least two comorbidities and 13% had cirrhosis. Once cirrhosis was excluded, the risk of any degree of fibrosis was 13.8% with NFS and 20.5% with FIB‐4 in subjects <65 years.

**Conclusions:**

Even if there is an identification gap in primary care, recorded cases with NAFLD have a high frequency of associated comorbidities. Despite regional variability, a close relation between cirrhosis and NAFLD exists (OR: 3.48, 95% CI: 3.23–3.76). Therefore, the use of non‐invasive tests should be promoted in primary care as a useful tool for the early identification of fibrosis risk, independently of evidence of steatosis.

**Lay Summary**

- Forecast epidemiological data suggest that the prevalence of NAFLD of 20–30% in the general population will cause a growing incidence of cirrhosis and liver cancer.
- The presence of NAFLD was observed in 9% of subjects in primary care; this finding underlines a registration gap likely owing to non‐invasive diagnostic difficulties and patient identification.
- The presence of fibrosis is the main driver of hepatic and extra‐hepatic complications. Moreover, the presence of fibrosis in NAFLD may remain undiagnosed, reducing treatment options because several physicians are unaware of non‐invasive tests (NITs) based on a simple combination of laboratory results other than liver biopsy for stratifying the risk of liver fibrosis.
- This is the first study to evaluate the risk of fibrosis in Italian primary care. We provide evidence that strongly suggests identifying NAFLD subjects at risk for fibrosis.
- The findings suggest that using NITs in primary care services may optimize the appropriate referral of NAFLD subjects at risk for fibrosis.

## 1 BACKGROUND

Non‐alcoholic fatty liver disease (NAFLD) is characterized by the excessive accumulation of triglycerides in the liver. It is often associated with insulin resistance and metabolic syndrome (MetS) with common clinical manifestations, such as hypertension, dyslipidemia, visceral adiposity and glucose intolerance. NAFLD is considered the most common cause of liver injury, with a prevalence exceeding 20% in the general population.^1^

NAFLD's ‘global burden’ is linked to its potential evolution through inflammation into non‐alcoholic steatohepatitis (NASH) and fibrosis. Fibrosis is the determining factor in the natural history of NAFLD. Fibrosis is significantly associated with mortality and morbidity from cardiovascular diseases, extra‐hepatic cancer and major hepatological events (i.e., cirrhosis with its complications and hepatocellular carcinoma).^2^ Furthermore, the early identification of patients with significant fibrosis within a population at risk and the correct management of precipitating factors may reduce the increasing request for liver transplantation owing to cirrhosis secondary to NASH.^3^

Despite the great impact of fibrotic liver disease on the health systems, healthcare costs and quality of life,^4^ no effective treatment has been approved for the management of NASH. The current prevalence of NAFLD and NASH in Italy is unknown.^5^, ^6^, ^7^ Therefore, the early diagnosis of NASH is a highly recommended strategy.^8^, ^9^, ^10^, ^11^ Several NITs have been developed as an alternative to biopsy for diagnosing fibrotic NASH. In almost all the guidelines, only two NITs have been recommended for diagnosing NAFLD in subjects requiring liver consultation: the NAFLD fibrosis score (NFS) and the FIB‐4 index.^12^ Both can help general practitioners (GPs) to identify patients who need to be referred to a specialist for better stratification of liver disease.

Although NAFLD represents an emerging problem with a high impact on healthcare systems, it has been reported that many countries have not prepared specific plans or models of care for this chronic condition^13^, ^14^ and that there is a diagnostic/registration gap in primary care in Europe.^6^

Our study aimed to determine the prevalence of NAFLD and the probability of fibrosis using NITs in the Italian adult primary care service. The secondary endpoints were the assessment of comorbidities and the predicting factors of NAFLD.

## 2 MATERIALS AND METHODS

This is a retrospective study with de‐identified cases. The Ethical Committee of Fondazione Policlinico Gemelli IRCCS approved the study (Study ID 51634, protocol no. 2545/19).

### 2.1 Data source

Data were gathered from the Health Search Database (HSD), created for research purposes by the Italian College of General Practitioners and Primary Care. The HSD has data from approximately 1 million individuals registered anonymously and voluntarily by a network of about 1000 GPs. These data are representative of each geographic macro area in terms of the number of reference populations. This data source contains the patient demographics, linked with an encrypted code to clinical records (i.e., diagnoses, procedures and medications), coded using the 9th International Classification of Diseases Revision Clinical Modification (ICD‐9‐CM), and drug prescriptions coded using the Anatomical Therapeutic Chemical classification system. This database fulfils the standard quality criteria and has been previously used for various epidemiological purposes.^6^, ^15^, ^16^

### 2.2 Study population

All individuals aged 18 years or older with at least 1 year of clinical history in the database from 2008 to 2017 were included in the study.

### 2.3 Case definition

Patients with NAFLD were identified through the ICD‐9‐CM diagnostic code (571.8)^17^ and the Hepatic Steatosis Index (HSI) >36,^18^ calculated using the last measured parameters. The date of diagnosis of NAFLD was considered the study index date.

The HSI score was obtained using the following formula:

$$\begin{array}{c} \mathrm{HSI}=8^{*}\text{alanine aminotransferase}\;\left(\mathrm{ALT}\right)/\text{aspartate aminotransferase}\;\left(\mathrm{AST}\right)\\ \;+\mathrm{BMI}\;\left(+2\;\text{if type}\;2\;\text{diabetes},+2\;\text{if female}\right). \end{array}$$

A value of >36 was used to rule in steatosis and validated in the Italian population.^18^, ^19^

All cases with a history of alcohol abuse or alcohol‐related diseases, hepatitis B or C infection, autoimmune liver diseases, primary biliary cholangiopathy or hepatic or extra‐hepatic neoplasia before or on the index date were excluded. Details on the exclusion algorithm were reported in Table S1.

### 2.4 Study population characteristics

The following data were retrieved for each patient: date of birth, age, gender, height, weight, blood pressure and laboratory data (transaminases, platelet counts, glycaemia, glomerular filtration rate [GFR]) assessed 2 years before or 6 months after the index date. The most recent results recorded in 2017 were considered.

Comorbidities, such as diabetes, MetS, cerebro‐cardiovascular diseases, congestive heart failure and HIV, were identified using the ICD‐9‐CM codes (Table S2). Charlson Comorbidity Index has been used for comorbidities.

Cirrhosis was identified using the ICD‐9‐CM codes (before or after the index date), and/or the presence of oesophageal varices was identified using the ICD‐9‐CM codes (456.0 and 456.1).

The severity of chronic kidney disease was assessed using the KDIGO guidelines: normal (GFR > 90 ml/min/1.73 m^2^), mild (GFR 60–89 ml/min/1.73 m^2^), moderate (GFR 30–59 ml/min/1.73 m^2^), severe (15–29 ml/min/1.73 m^2^) and end‐stage renal disease or kidney failure if the GFR <15 ml/min/1.73 m^2^ or the individual scheduled for ‘dialysis’ or ‘renal transplantation’ in the database.

Polypharmacy was defined as the regular use of at least five concurrent categories according to the WHO Anatomical Therapeutic Chemical Classification System,^20^ prescribed within 3 months before or after the index date.

### 2.5 Liver fibrosis assessment

The FIB‐4 and NFS were used to assess the probability of the presence of liver fibrosis.^21^, ^22^, ^23^

The scores were calculated as follows:

$$\mathrm{FIB}-4=\mathrm{age}\;\left(\left[\text{years}\right]\;x\;\mathrm{AST}\;\left[U/L\right]\right)/\left(\left(\mathrm{PLT}\;\left[10^{9}/L\right]\right)\;x\;\left(\mathrm{ALT}\;\left[U/L\right]\right)\;\left(1/2\right)\right)$$

$$\begin{array}{c} \mathrm{NFS}=-1.675+0.037\times \mathrm{age}\;\left(\text{years}\right)+0.094\times \mathrm{BMI}\;\left(\mathrm{kg}/m^{2}\right)\\ \;+1.13\times \mathrm{IFG}/\text{diabetes}\;\left(\mathrm{yes}=1,\mathrm{no}=0\right)+0.99\times \mathrm{AST}/\mathrm{ALT}\;\text{ratio}-0.013\\ \;\times \text{platelet}\;\left(\times 109/l\right)-0.66\times \text{albumin}\;\left(g/\mathrm{dl}\right) \end{array}$$

Since the scores are influenced by age, subjects were divided into two groups: those under 65 years old and those 65 years or older.

The probability of advanced fibrosis in the younger subjects was defined as low (FIB‐4 < 1.3 or NFS < ‐1.455), intermediate (FIB‐4: 1.3–2.67 or NFS: −1.455‐0.672) and high (FIB‐4 > 2.67 or NFS >0.672). Moreover, for the FIB‐4 score, we used a cut‐off of >3.25 to identify the cases with a higher probability of advanced fibrosis.^12^

Age‐adjusted scores were used for subjects aged 65 years and older. A low probability of developing fibrosis was considered when the FIB‐4 was <2 or NFS was <−0.12.^21^

### 2.6 Nested case–control analysis

The first cases of NAFLD identified in the eligibility period were matched to 10 controls for age (±5 years), sex, the database's entry date and the follow‐up duration. This design allowed us to estimate the determinants of NAFLD.

### 2.7 Data analyses

Descriptive statistics were carried out. Continuous and categorical variables were presented as means (standard deviation, SD), medians, interquartile ranges or absolute numbers and percentages, as appropriate. The prevalence of NAFLD in 2017 was calculated as the ratio of the number of cases divided by the total active HSD subjects in the same year, considering all cases, and subsequently stratified by gender, age, or region of residence. Trends of NAFLD incidence and prevalence during 2008–2017 were considered.

Differences in the demographic and clinical characteristics between the cases and controls were analysed using the t test. The chi‐square was used to test for between‐group differences among the continuous and categorical variables. A conditional logistic model was adopted to estimate the univariate and multivariate odds ratio (OR) and related 95% CI for each determinant of NAFLD. No imputation was provided for the missing values in the categorical variables and they were considered a separate category in each model. Analyses were performed using Stata v.13.0.

## 3 RESULTS

### 3.1 Characteristics of patients diagnosed with NAFLD

The prevalence of NAFLD among the active patients undergoing primary care (i.e., alive and registered in the GPs' lists) in HSD in 2017 is shown in Table 1. Among the 918 954 active individuals, 83 981 (9.14%) had NAFLD, 18309 were identified using the ICD‐9‐CM diagnostic code, and 65 672 had an HSI score of >36. (Table S3 and S4).

**TABLE 1:** Prevalence rate of non‐alcoholic fatty liver disease in Italian primary care patients in 2017

	Female			Male			Total		
	Active population (n)	NAFLD cases (n)	Prevalence (%)	Active population (*n*)	NAFLD cases (*n*)	Prevalence (%)	Active population (*n*)	NAFLD cases (*n*)	Prevalence (%)
Total	481 099	39 274	8.16	437 855	44 707	10.21	918 954	83 981	9.14
Age (years)									
18–27	53 335	1440	2.7	57 712	1430	2.48	111 047	2870	2.58
28–37	65 628	2698	4.11	65 446	3194	4.88	131 074	5892	4.5
38–47	82 899	4613	5.56	77 649	7173	9.24	160 548	11 786	7.34
48–57	89 838	6940	7.73	81 396	10 844	13.32	171 234	17 784	10.39
58–67	69 967	8226	11.76	64 670	10 341	15.99	134 637	18 567	13.79
68–77	58 856	8520	14.48	52 332	7861	15.02	111 188	16 381	14.73
78–87	44 809	5542	12.37	31 457	3440	10.94	76 266	8982	11.78
≥88	15 767	1295	8.21	7193	424	5.89	22 960	1719	7.49
Italian regions									
Abruzzo	14 669	1533	10.45	13 096	1777	13.57	27 765	3310	11.92
Basilicata	7002	533	7.61	6909	748	10.83	13 911	1281	9.21
Calabria	14 430	1423	9.86	13 454	1376	10.23	27 884	27,99	10.04
Campania	52 781	6072	11.5	47 451	6064	12.78	100 232	12 136	12.11
Emilia‐Romagna	23 761	1252	5.27	20 254	1851	9.14	44 015	3103	7.05
Friuli‐Venezia Giulia	27 706	2366	8.54	25 110	2776	11.06	52 816	5142	9.74
Lazio	37 823	1800	4.76	32 368	2118	6.54	70 191	3918	5.58
Liguria	13 890	1062	7.65	12 193	1166	9.56	26 083	2228	8.54
Lombardia	76 943	6258	8.13	71 805	7891	10.99	148 748	14 149	9.51
Marche	12 256	666	5.43	11 510	814	7.07	23 766	1480	6.23
Piemonte	35 057	1178	3.36	32 447	1574	4.85	67 504	2752	4.08
Puglia	36 705	4233	11.53	34 324	4109	11.97	71 029	8342	11.74
Sardegna	13 049	1012	7.76	12 231	1122	9.17	25 280	2134	8.44
Sicilia	43 531	4232	9.72	40 035	4478	11.19	83 566	8710	10.42
Toscana	24 339	2422	9.95	22 210	2802	12.62	46 549	5224	11.22
Trentino‐Alto Adige	4334	94	2.17	3949	160	4.05	8283	254	3.07
Umbria	14 968	1215	8.12	13 064	1512	11.57	28 032	2727	9.73
Valle d'Aosta	635	55	8.66	513	47	9.16	1148	102	8.89
Veneto	27 220	1868	6.86	24 932	2322	9.31	52 152	4190	8.03

Abbreviation: NAFLD, non‐alcoholic fatty liver disease.

**FIGURE 1:**
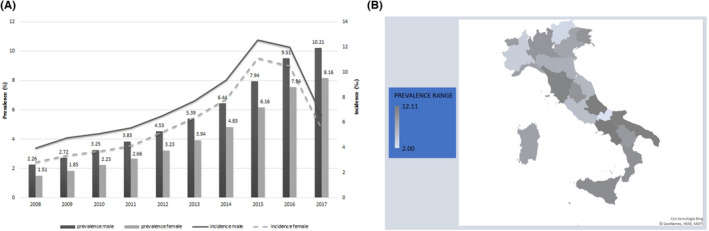
Prevalence and incidence of NAFLD according to sex (A) and variability of NAFLD prevalence rate among Italian regions (B).

NAFLD was higher present in male than in female subjects (10.2 vs. 8.2%) and in the older group: 2.6% (18–27 years) and 14.7% (68–77 years). Concerning the region of residence, a certain degree of variability was observed (Figure 1). The lowest presence of NAFLD was found in Trentino‐Alto Adige (3.1%), while the highest was in Campania (12.1%). An increasing trend in the prevalence of NAFLD was observed in 2018–2017, while the incidence increased until 2015 and then decreased (Table S2).

The clinical and demographic characteristics of NAFLD cases identified during the eligibility period (2008–2017) are reported in Table 2. Overall, 151 431 subjects with NAFLD were identified, and 5.6% stemmed from the direct ICD‐9‐CM coding system. Most of them were 38–67 years old, and the prevalence decreased after the age of 68 years.

**TABLE 2:** Characteristics of non‐alcoholic fatty liver disease cases in the Italian General Practitioners' cohort

Description	*n* (%)/mean ± *SD*
Subjects with NAFLD	151 431
ICD‐9‐CM	8449 (5.6)
Hepatic Steatosis Index >36	142 982 (94.4)
Gender	
Female	74 573 (49.25)
Male	76 858 (50.75)
Age (years)	57.15 ± 15.74
Age groups	
18–27	6243 (4.12)
28–37	12 343 (8.15)
38–47	23 070 (15.23)
48–57	31 022 (20.49)
58–67	35 839 (23.67)
68–77	28 835 (19.04)
78–87	12 558 (8.29)
88+	1521 (1.00)
Follow‐up (months)	33.19 ± 28.63
BMI (kg/m^2^)	29.44 ± 6.82
Obesity	
BMI ≤30	71 237 (47.04)
BMI >30	42 314 (27.94)
NA	37 880 (25.01)
Blood pressure (mmHg)	
Systolic blood pressure	134.04 ± 16.97
Diastolic blood pressure	81.34 ± 9.53
Comorbidities	
Diabetes	37 413 (24.71)
Metabolic syndrome	3329 (2.2)
Cerebro/cardiovascular disease	16 632 (10.98)
Heart failure	6669 (4.4)
HIV	146 (0.1)
Kidney function (GFR)	
Normal (≥90)	25 381 (16.76)
Mild (60–89)	110 882 (73.22)
Moderate (30–59)	14 379 (9.5)
Severe (15–29)	429 (0.28)
Kidney failure (<15)	360 (0.24)
Charlson comorbidities	
0	18 194 (12.01)
1	5116 (3.38)
2	83 496 (55.14)
>2	44 625 (29.47)
Polypharmacy (concurrent medications)	
<5	67 997 (44.9)
5+	66 181 (43.7)
No therapy	17 253 (11.39)
Liver cirrhosis	20 022 (13.22)
AST/ALT	0.9 ± 0.51
Patients aged ≤65 years	
FIB‐4	1.1 ± 2.02
NFS	−3.12 ± 4.52
Patients aged >65 years	
FIB‐4	1.91 ± 2.18
NFS	−1.59 ± 1.83

Abbreviations: ALT, aminotransferase; AST, aspartate aminotransferase; BMI, body mass index; GFR, glomerular filtration rate; NA, not available; NFS, NAFLD fibrosis score.

Among the cases with NAFLD, 28% were obese; 29.5% had two or more comorbidities; 43.7%, polytherapy; 24.7%, diabetes; 2.2%, MetS; 11%, cerebro‐cardiovascular disease; 4.4%, hearth failure; and 0.1%, HIV. Moderate or severe kidney disease was diagnosed in 10% of subjects with NAFLD.

Cirrhosis was diagnosed in 13.2% of patients with NAFLD undergoing primary care.

The AST/ALT ratio was not available for only 2736 cases and was higher than 1.0 in 27.7% of cases with NAFLD.

**FIGURE 2:**
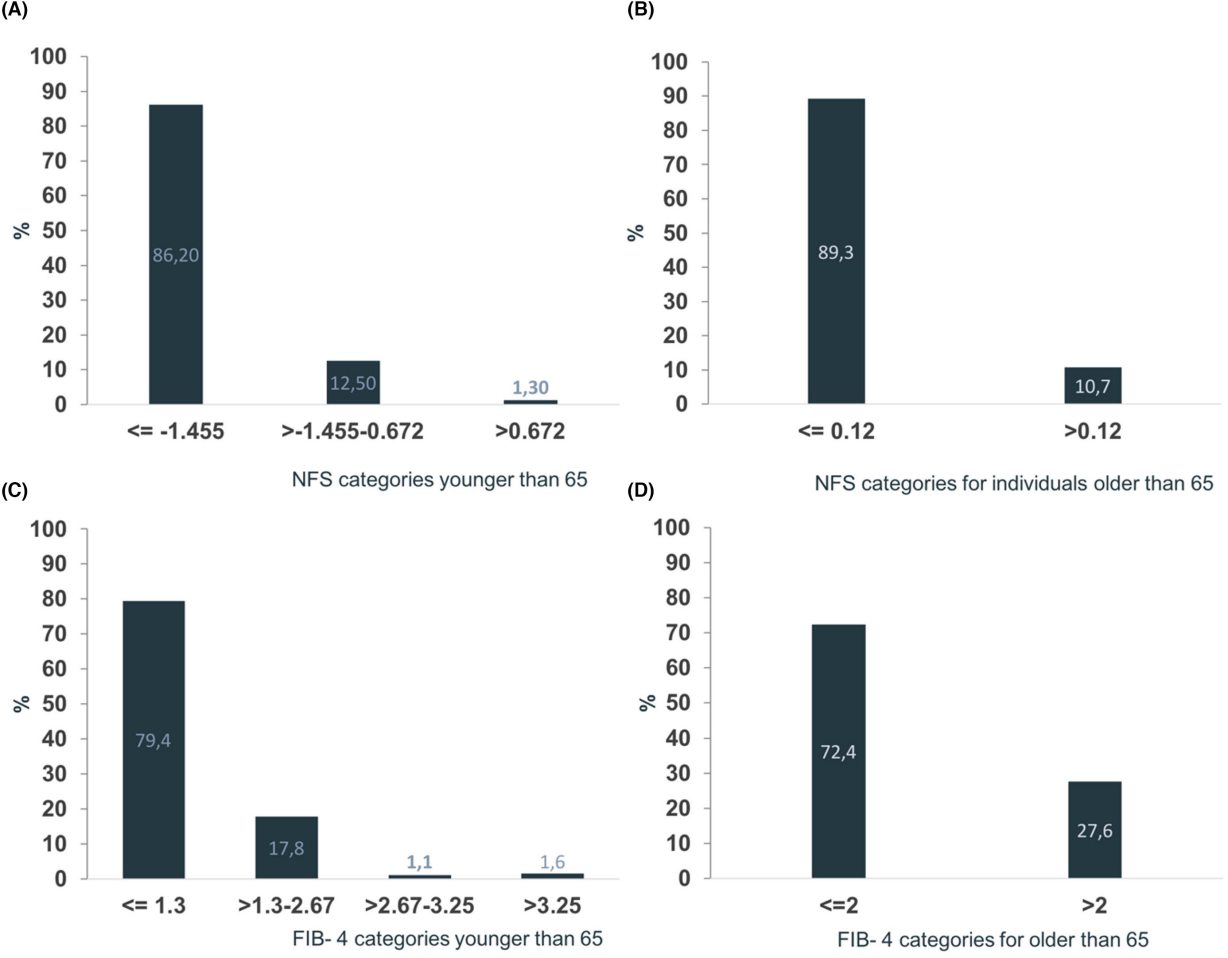
Probability of fibrosis according to NAFLD fibrosis score (NFS) and FIB‐4. Percentage of subjects at low, intermediate and high risk of fibrosis. NFS distribution in individuals (A) younger than 65 years and (B) older than 65 years. FIB‐4 distribution individuals (C) younger than 65 years and (D) older than 65 years.

The probability of fibrosis varied according to the NIT used. For subjects younger than 65 years, the probability of fibrosis was 13.8% with NFS and 20.5% with FIB‐4, while for the older population, it was 10.7% with NFS and 27.6% with FIB‐4 (Figure 2).

### 3.2 Comparison of the clinical characteristics of the NAFLD cases and matched controls

**FIGURE 3:**
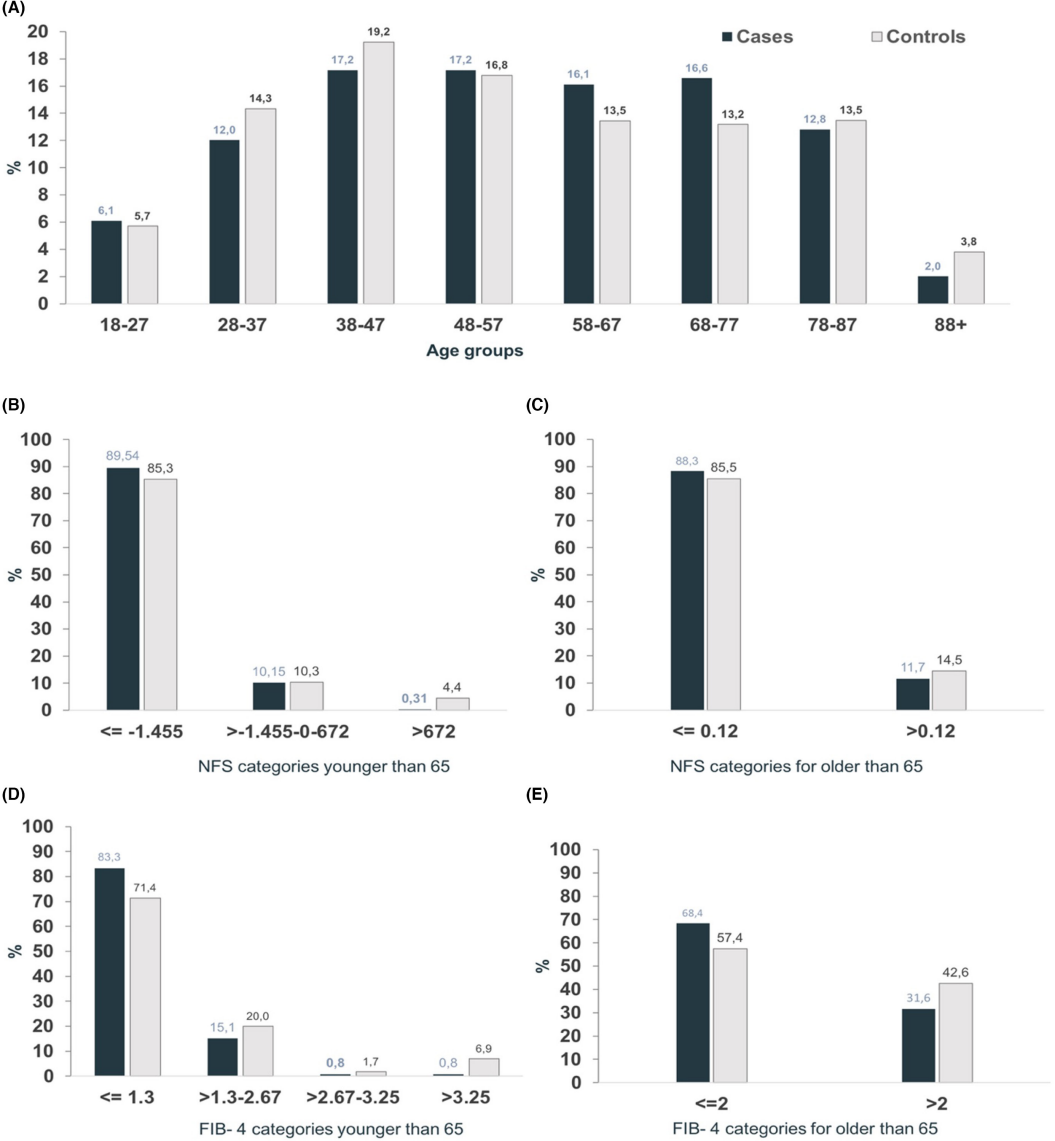
Distribution of non‐alcoholic fatty liver disease (NAFLD) subjects according to age and fibrosis index categories. Distribution of individuals according to (A) age groups, (B) NAFLD fibrosis score (NFS) in younger than 65 years and (C) NFS in older than 65 years, (D) FIB‐4 in younger than 65 years and (E) FIB‐4 in older than 65 years.

Cases with NAFLD (*n* = 67 747) were matched to 252 991 controls (Table 3) across all ages (Figure 3A).

**TABLE 3:** Characteristics of the non‐alcoholic fatty liver disease cases and related controls

Description	Cases (*n* = 67 747), *n* (%)/mean ± *SD*	Controls (*n* = 252 991), *n* (%)/mean ± *SD*
Diagnosis of NAFLD		
ICD‐9‐CM	4628 (6.83)	
HSI	63 119 (93.17)	
Follow‐up (months)	45.07 ± 31.05	51.18 ± 32.68
Gender		
Female	32 177 (47.5)	118 510 (46.8)
Male	35 570 (52.5)	134 481 (53.2)
Age (years)	56.13 ± 18.19	55.54 ± 19.04
Age groups		
18–27	4133 (6.1)	14 419 (5.7)
28–37	8152 (12.03)	36 283 (14.34)
38–47	11 631 (17.17)	48 666 (19.24)
48–57	11 637 (17.18)	42 482 (16.79)
58–67	10 923 (16.12)	34 032 (13.45)
68–77	11 235 (16.58)	33 349 (13.18)
78–87	8676 (12.81)	34 099 (13.48)
88+	1360 (2.01)	9661 (3.82)
BMI	29.01 ± 7.4	25.59 ± 6.84
Obesity		
BMI ≤30	33 316 (49.18)	72 600 (28.7)
BMI >30	16 692 (24.64)	13 647 (5.39)
NA	17 739 (26.18)	166 744 (65.91)
Blood pressure (mmHg):		
Diastolic blood pressure	80.6 ± 9.72	78.56 ± 9.84
Systolic blood pressure	132.66 ± 17.13	129.95 ± 18.92
Comorbidities		
Diabetes	6472 (9.55)	21 220 (8.58)
Metabolic syndrome	913 (1.35)	440 (0.17)
Cerebro/cardiovascular disease	6343 (9.36)	11 786 (4.66)
Heart failure	2968 (4.38)	7272 (2.87)
HIV	73 (0.11)	290 (0.11)
Cirrhosis	8850 (13.06)	6344 (2.51)
Kidney functional impairment (GFR):		
Normal (≥90)	12 061 (17.8)	145 154 (57.38)
Mild (60–89)	48 485 (71.57)	88 072 (34.81)
Moderate (30–59)	6814 (10.06)	17 061 (6.74)
Severe (15–29)	224 (0.330	1539 (0.61)
Kidney failure (<15)	163 (0.24)	1165 (0.46)
Charlson Index		
0	8826 (13.03)	116 954 (46.23)
1	2416 (3.57)	17 557 (6.94)
2	36 830 (54.36)	62 051 (24.53)
>2	19 675 (29.04)	56 429 (22.3)
Polypharmacy (concurrent medications)		
<5	31 640 (46.7)	31 246 (12.35)
5+	27 062 (39.95)	33 222 (13.13)
No therapy	9045 (13.35)	188 523 (74.52)
Level of fibrosis		
Raised ALT (UNL: 19 UI/L for female and 30 UI/L for male)	38 917 (58.89)	22 637 (38.42)
AST/ALT	0.9 ± 0.41	1.2 ± 0.8
Subjects aged ≤65 years:		
FIB‐4	0.93 ± 0.58	1.52 ± 2.42
NFS	−3.57 ± 7.23	−5.29 ± 15.63
Subjects aged >65 years:		
FIB‐4	2.07 ± 2.59	2.65 ± 3.73
NFS	−1.39 ± 1.62	−1.49 ± 2.16

*Note*: Cases identified via ICD9‐CM.

Abbreviations: ALT, aminotransferase; AST, aspartate aminotransferase; BMI, body mass index; GFR, glomerular filtration rate; NA, not available; NFS, NAFLD fibrosis score.

The BMI (as a continuous variable and obesity: BMI >30) was greater among the cases than in controls, while there was no difference in systolic and diastolic blood pressure. Except for HIV, the proportion of comorbidities was higher among the cases than in controls. The presence of cirrhosis and the complications of liver diseases were significantly higher among the cases than in controls (13.1% vs. 2.5%, *p* < .001). The proportion of those with renal functional impairment, including kidney failure, was higher in the cases with NAFLD than in controls. Greater values for Charlson Index were also observed.

The probability of fibrosis varied according to the NITs used. The probability of fibrosis was intermediate–high in 10.46% of NAFLD cases and 14.7% of controls in subjects aged <65 years (Figure 3B) and 11.7% of NAFLD cases and 14.7% of controls in subjects aged >65 years (Figure 3C). In the same population, the FIB‐4 identified that 16.7% of NAFLD cases aged <65 had an intermediate–high risk of fibrosis compared with 28.5% of controls (Figure 3D). In subjects aged >65 years, the probability of fibrosis was 31.6% in NAFLD cases and 42.6% in controls using the FIB‐4 (Figure 3E).

### 3.3 Factors associated with the presence of NAFLD

When the conditional logistic regression was used (Table 4), obesity had a higher adjusted OR (aOR: 2.3, 95% CI: 2.49–2.64) in the NAFLD group than in related controls. Blood pressure did not show any association with NAFLD occurrence. MetS (aOR; 2.3, 95% CI: 1.9–2.8), cerebro/cardiovascular disease (aOR: 1.4, 95% CI: 1.3–1.5), heart failure (aOR: 1.1, 95% CI: 1–1.2) and liver complications, such as cirrhosis (aOR: 3.7, 95% CI: 3.5–4), were significantly associated with the odds of having NAFLD. The risk of NAFLD increased according to the severity of kidney disease. Patients with a very low GFR (<15) had a 6.7‐fold greater risk of NAFLD than subjects with normal kidney function.

**TABLE 4:** Determinants of non‐alcoholic fatty liver disease occurrence

	Overall			Age < 65 years			Age ≥ 65 years		
	Crude OR	AOR (95% CI)	AOR^a^ (95% CI)	Crude OR	AOR^b^ (95% CI)	AOR^a^ ^,^ ^b^ (95% CI)	Crude OR	AOR^b^ (95% CI)	AOR^a^ ^,^ ^b^ (95% CI)
Obesity									
BMI ≤30 (reference)	–	–	–	–	–	–	–	–	–
BMI >30	2.56 (2.49–2.64)	2.28 (2.18–2.39)	–	2.88 (2.77–3)	3.14 (2.76–3.58)	–	2.27 (2.17–2.38)	2.98 (2.72–3.26)	–
NA	0.21 (0.21–0.22)	0.39 (0.37–0.41)	–	0.17 (0.17–0.18)	0.33 (0.29–0.36)	–	0.32 (0.31–0.33)	0.41 (0.38–0.44)	–
Blood pressure (mmHg)									
Diastolic pressure	1.02 (1.02–1.02)	1.01 (1.01–1.01)	1.01 (1.01–1.01)	1.03 (1.03–1.03)	1.02 (1.01–1.02)	1.02 (1.01–1.02)	1.01 (1.01–1.01)	1.01 (1.01–1.01)	1.01 (1.01–1.01)
Systolic pressure	1.01 (1.01–1.01)	1.01 (1.01–1.01)	1.01 (1.01–1.01)	1.02 (1.02–1.02)	1.01 (1.01–1.01)	1.01(1.01–1.01)	1.01 (1.01–1.01)	1.01 (1.01–1.01)	1.01 (1.01–1.01)
Comorbidities									
Metabolic syndrome	6.61 (5.85–7.47)	2.29 (1.88–2.8)	3.35 (2.77–4.06)	9.35 (7.87–11.11)	1.73 (1.07–2.81)	2.85 (1.82–4.46)	4.45 (3.68–5.4)	2.07 (1.43–2.98)	3.59 (2.54–5.09)
Cerebro/cardiovascular disease	2.04 (1.96–2.11)	1.37 (1.29–1.45)	1.39 (1.32–1.47)	4.22 (3.9–4.57)	1.48 (1.16–1.87)	1.59 (1.27–1.99)	1.65 (1.58–1.72)	1.29 (1.19–1.4)	1.32 (1.22–1.42)
Heart failure	1.47 (1.4–1.54)	1.09 (1.02–1.17)	1.17 (1.1–1.26)	1.82 (1.54–2.15)	0.74 (0.46–1.2)	0.87 (0.56–1.35)	1.44 (1.37–1.52	1.15 (1.04–1.27)	1.24 (1.13–1.36)
HIV	0.96 (0.73–1.26)	0.91 (0.5–1.63)	0.84 (0.47–1.5)	0.91 (0.68–1.2)	0.64 (0.28–1.44)	0.58 (0.26–1.27)	1.92 (0.74–4.99)	0.71 (0.07–7.07)	0.8 (0.08–7.8)
Cirrhosis	5.94 (5.72–6.16)	3.73 (3.49–3.99)	3.42 (3.2–3.64)	11.38 (10.78–12.01)	15.38 (12.97–18.24)	13.22 (11.25–15.53)	2.72 (2.57–2.88)	3.33 (2.98–3.72)	2.93 (2.64–3.25)
Kidney function (GFR)									
Normal (≥90)	–	–	–	–	–	–	–	–	–
Mild (60–89)	2.22 (1.86–2.65)	1.98 (1.48–2.66)	1.93 (1.45–2.56)	3.32 (2.54–4.33)	2.72 (1.14–6.47)	2.11 (0.92–4.83)	1.26 (0.98–1.62)	0.63 (0.37–1.06)	0.58 (0.36–0.94)
Moderate (30–59)	2.69 (2.31–3.13)	1.9 (1.49–2.43)	1.99 (1.58–2.52)	3.21 (2.08–4.95)	0.4 (0.1–1.63)	0.43 (0.12–1.5)	1.95 (1.65–2.3)	0.59 (0.41–0.86)	0.59 (0.42–0.83)
Severe (29–15)	7.31 (7.01–7.62)	5.72 (5–6.54)	5.92 (5.21–6.72)	9.11 (8.35–9.95)	3.11 (1.97–4.91)	2.99 (1.94–4.62)	5.18 (4.87–5.5)	1.37 (1.07–1.76)	1.34 (1.06–1.68)
End‐stage CKD (<15)	8.29 (8.08–8.51)	6.71 (5.9–7.63)	6.78 (6–7.66)	9.19 (8.92–9.48)	3.54 (2.34–5.34)	3.33 (2.25–4.91)	5.75 (5.45–6.06)	1.36 (1.07–1.74)	1.33 (1.06–1.66)
Charlson Comorbidity Index									
0 (reference)									
1	2.09 (1.98–2.2)	0.83 (0.75–0.93)	0.86 (0.78–0.96)	2.21 (2.08–2.34)	0.78 (0.61–1.01)	0.81 (0.65–1.03)	1.44 (1.28–1.61)	0.83 (0.62–1.12)	0.81 (0.62–1.06)
2	9.77 (9.48–10.06)	0.53 (0.46–0.61)	0.54 (0.47–0.61)	9.94 (9.61–10.27)	0.23 (0.15–0.35)	0.24 (0.16–0.35)	7.33 (6.83–7.87)	0.52 (0.39–0.71)	0.51 (0.38–0.67)
>2	6.62 (6.4–6.85)	0.23 (0.2–0.26)	0.24 (0.21–0.27)	8.71 (8.35–9.08)	0.11 (0.07–0.17)	0.11 (0.08–0.17)	3.99 (3.72–4.28)	0.19 (0.14–0.26)	0.19 (0.14–0.25)
Polypharmacy (concurrent medications)									
No therapy (reference)	–	–	–	–	–	–	–	–	–
<5	34.52 (33.2–35.88)	27.05 (25.41–28.78)	27.4 (25.8–29.11)	34.33 (32.87–35.85)	15.89 (14.22–17.75)	16.22 (14.62–17.99)	33.16 (30.2–36.41)	41.58 (35.1–49.24)	40.02 (34.01–47.09)
5+	46.33 (44.36–48.38)	37.85 (35.43–40.44)	38.68 (36.27–41.24)	45.92 (43.38–48.6)	18.34 (16.05–20.94)	18.48 (16.33–20.92)	45.54 (41.57–49.88)	55.63 (47.16–65.61)	53.86 (45.94–63.14)

Abbreviations: ALT, aminotransferase; AOR, adjusted OR; AST, aspartate aminotransferase; BMI, body mass index; CKD, chronic kidney disease; GFR, glomerular filtration rate; NA, not available; NFS, NAFLD fibrosis score; OR, odds ratio.

^a^ Excluding BMI.

^b^ Adjusted for NFS, FIB‐4, and ALT/AST.

Therefore, the increased Charlson Index associated with an unexpected protective effect may be ascribed to the strong associations observed for the covariates mentioned above. The concurrent use of five or more medications was significantly associated with NAFLD occurrence. Besides the BMI, multivariate analysis did not show any difference in the OR for the other cofactors considered.

When only the cohort of younger individuals was considered, the number of obese cases was significantly higher (aOR: 3.1, 95% CI: 2.8–3.6) among cases than in controls. MetS (aOR: 1.7, 95% CI: 1.1–2.8), cerebro/cardiovascular disease (aOR: 1.5, 95% CI; 1.2–1.9), end‐stage CKD (≤15) (aOR: 3.5, 95% CI: 2.3–5), and liver complications, such as cirrhosis (aOR:15.4, 95% CI: 13–1.2), were significantly associated with NAFLD.

We also performed a multivariable analysis matched by region (Table 5) to explain the variability observed. Indeed, NAFLD was associated with MetS (aOR: 4.44, 95% CI: 3.41–5.77), cerebro/cardiovascular disease (aOR: 1.48, 95% CI: 1.38–1.58) and heart failure (aOR: 1.2, 95% CI: 1.09–1.31) and cirrhosis (aOR: 3.48, 95% CI: 3.23–3.76). Even in this multivariable analysis, NAFLD has been associated with worsening kidney function and polypharmacy use.

**TABLE 5:** Determinants of non‐alcoholic fatty liver disease occurrence (matched by region)

	Overall		
	Crude OR	AOR (95% CI)	AOR^a^ (95% CI)
Obesity			
BMI ≤ 30 (reference)	–	–	–
BMI > 30	2.62 (2.53–2.71)	2.33 (2.2–2.47)	–
NA	0.21 (0.2–0.21)	0.39 (0.37–0.41)	–
Blood pressure (mmHg)			
Diastolic pressure	1.02 (1.02–1.02)	1.01 (1.01–1.02)	1.01 (1.01–1.02)
Systolic pressure	1.01 (1.01–1.01)	1.01 (1–1.01)	1.01 (1.01–1.01)
Comorbidities			
Metabolic syndrome	8.51 (7.31–9.91)	3.25 (2.47–4.27)	4.44 (3.41–5.77)
Cerebro/cardiovascular disease	2.26 (2.16–2.36)	1.48 (1.37–1.58)	1.48 (1.38–1.58)
Heart failure	1.49 (1.4–1.58)	1.11 (1.01–1.21)	1.2 (1.09–1.31)
HIV	0.83 (0.61–1.12)	0.64 (0.33–1.24)	0.54 (0.27–1.07)
Cirrhosis	6.24 (5.98–6.51)	3.86 (3.56–4.18)	3.48 (3.23–3.76)
Kidney function (GFR)			
Normal (≥90)	–	–	–
Mild (60–89)	1.57 (1.28–1.94)	1.52 (1.05–2.19)	1.68 (1.19–2.38)
Moderate (30–59)	2.32 (1.93–2.78)	1.75 (1.28–2.41)	1.73 (1.28–2.34)
Severe (15–29)	6.65 (6.34–6.98)	5.89 (5.02–6.9)	6.1 (5.25–7.09)
Kidney failure (<15)	7.87 (7.65–8.09)	7.14 (6.13–8.31)	7.28 (6.31–8.39)
Charlson Comorbidities Index			
0 (reference)			
1	1.93 (1.83–2.04)	0.75 (0.66–0.85)	0.8 (0.71–0.9)
2	8.98 (8.7–9.27)	0.51 (0.43–0.6)	0.51 (0.44–0.6)
>2	6.02 (5.81–6.24)	0.21 (0.18–0.25)	0.22 (0.19–0.26)
Polypharmacy (concurrent medications)			
No therapy (reference)	–	–	–
<5	29.75 (28.53–31.03)	25.31 (23.55–27.22)	25.95 (24.19–27.83)
5+	39.83 (37.96–41.79)	36.62 (33.85–39.61)	37.86 (35.09–40.86)

*Note*: Non‐alcoholic fatty liver disease cases *n* = 62 025 and controls *n* = 16 395.

Abbreviations: AOR, adjusted OR; BMI, body mass index; GFR, glomerular filtration rate; NA, not available; OR, odds ratio.

a BMI is excluded.

## 4 DISCUSSION

The epidemiological models developed on NAFLD suggest that the incidence of liver‐related complications will double in the next few years.^24^ To our knowledge, this is the first study investigating the burden of NAFLD in the Italian primary care setting using real‐world data. More than 94% of cases with NAFLD were identified using the HSI, which had been previously indicated as a predictor of NAFLD in the Italian population.^18^, ^19^ This observation shows that many cases go undetected and/or unreported, which could be ascribed to the lack of a specific ICD‐9‐CM code, thus placing GPs in a difficult position on how to record the condition and, consequently, how to best identify patients.^25^

In recent years, scientific societies have focused on the awareness of NAFLD, especially in subjects with simple steatosis at high risk of progression to NASH because of the need for close monitoring and urgent therapeutic intervention. Owing to confusion over the term non‐alcoholic,^11^ recently, a panel of experts suggested renaming NAFLD with metabolic dysfunction‐associated fatty liver disease,^26^ a term that could enhance attention to liver diseases associated with metabolic conditions and the need for NITs in monitoring these patients over time.^27^

From our study, the presence of NAFLD was in 9% of the active population in primary care in 2017, with significant regional variability that could be partially explained by social and cultural differences in diet and lifestyle. This variability was present in other studies on other chronic diseases.^28^

Historical data on the prevalence of fatty liver in Italy from the Dionysos study are from a general population cohort in a different historical period, with different lifestyles and in the age group 12–65 years, and from an Italian regional district.^29^ To the best of our knowledge, our study represents the first epidemiological study in Italian primary care services and also focuses on regional variability.

The low prevalence (9%) confirms the identification/registration gap at the primary care level. This phenomenon seems to be related not only to the low awareness of NAFLD; however, it may be ascribed to structural elements, such as the lack of the disease‐specific code and the failure or partial recording in the electronic database of the data of patients who did not consent to share anonymized data for the calculation of the diagnosed indices above as well as the lack of recording of imaging parameters and liver function tests. The lack of data entry is certainly influenced by the low confidence in primary care in diagnosing NAFLD, which, in the absence of a specific code, is still not perceived as an important disease with the potential risk of evolution. Of course, it should be considered that guidelines on using NITs to identify cases at risk of fibrosis have only recently been published in Italy.^30^ The progressive increase in the prevalence and incidence of NAFLD cases may be explained by the increased primary care physicians' focus on liver disease in programs to identify HCV cases for treatment with direct antiviral drugs. The reduction in incidence could be explained by the stable population in primary care. The database used in primary care is unable to identify individuals with NASH who are likely to be included in NAFLD diagnosis. The diagnosis of NASH is based on the finding of steatosis, inflammation and ballooning on histological examination of liver biopsy. The absence of an administrative code and specific therapy probably further reduces the possibility of identifying cases in primary care.

The prevalence of NAFLD increased with age up to 78 years. This might be explained by the increased hospitalization rate or institutionalization of subjects who no longer receive GP consultations. A similar trend was observed in women, whereas in men, the prevalence increased up to 67 years. Until this age, the overall prevalence was higher in men and then became greater in women.

More than half of NAFLD patients had at least two coexisting chronic conditions. Diabetes (24.7%) and cerebrovascular and/or cardiovascular diseases (11%) were the most prevalent conditions identified respectively. These results are consistent with the findings from EU cohorts, and diabetes appears to have a significantly higher prevalence in NAFLD patients than in the general Italian population (8.3%). Our findings confirm recent observations on the UK biobank, which evaluated non‐invasively that steatosis is associated with a higher risk of major cardiovascular events.^31^ MetS is associated with NAFLD, confirming the importance of insulin resistance as the underlying causal factor in the absence of diabetes.

We obtained a correlation between severely impaired kidney function and NAFLD, as recently reported in a large meta‐analysis^32^ and a registry study where a reduction in the GFR was associated with an increase in FIB‐4 and NFS values.^33^

The high proportion of patients with polypharmacy was not surprising, as patients with NAFLD had concomitant comorbidities, especially those older than 65 years.^34^ Since using several drugs may cause additional liver injury, special attention should be paid when prescribing a new drug to a patient with NAFLD.

We obtained a high prevalence of advanced compensated liver disease and/or compensated cirrhosis (13%) in cases with NAFLD. This implies that an urgent, specific check and the promotion of actions devoted to the early identification of patients at risk of fibrosis using NITs is required, as scientific societies have already suggested.^27^ The use of NITs in primary care may help GPs identify NAFLD subjects with significant fibrosis, especially those <65 years, requiring in‐depth evaluation by specialistic consultation. In fact, our results, which align with those of other studies conducted in a primary care setting, revealed that 20.5% of NAFLD subjects had an intermediate–high probability of fibrosis that would require more specific clinical investigation.^27^ More interestingly, the probability of advanced fibrosis in the non‐NAFLD population aged <65 years varied between 4.4% with NFS and 8.6% with FIB‐4. The percentage of advanced fibrosis was increased in people >65 years; this could be linked to the inclusion of age in the algorithm of NFS and FIB‐4, as there is probably an underestimation of subjects with NAFLD in the control group. Certainly, the FIB‐4 and NFS approach to a primary care population may overestimate the risk of fibrosis. A recent international multicentre analysis documented that the use of FIB‐4 and NFS may be associated with increased false positives in the general population at low risk of fibrosis.^35^ The proportion of false positives was as high as one‐third of the study population, particularly in subjects aged >65. The proportion of false positives raised up to 30%, mainly in subjects aged >65.^35^

The increased proportion of fibrosis is responsible for both the progression of the disease and the increased mortality.^36^ Approximately 40% of NAFLD subjects with fibrosis may progress to more severe forms, as observed in long‐term follow‐up studies.^37^ As reported in the literature, it should be emphasized that NITs have prognostic significance for both cardiovascular events and progression to cirrhosis and hepatocellular carcinoma (HCC).^38^

Our study has some limitations. First, it was a retrospective study. Second, the ultrasonography diagnosis of fatty liver was not always available in the database. However, cases were identified using the ICD‐9‐CM code and validated his cut‐off of 36. Owing to the study's retrospective nature, we cannot exclude unrecognized conditions of viral hepatitis from HCV or HBV. Chronic viral hepatitis is registered in the primary care database because it has a unique identification code, and the focus in the study period to identify cases to be linked to care for initiation of antiviral therapies has been incremental since 2015 in primary care in Italy after the new direct antiviral drugs became available for all patients. Another weakness of the study is certainly the determination of alcohol consumption. Although no quantitative score is applied, at the time of data collection for the primary care database, alcohol consumption was collected based on self‐report and family member testimony and was declared inappropriate if it exceeded 20 g per day. Third, data on FIB‐4 and NFS were unavailable in the whole population, which may be explained by the fact that assessment and/or recording of liver biochemical tests are often confined only to subjects with abnormal liver function. Moreover, NITs usually overestimate the risk of fibrosis in elderly subjects. Therefore, it should be used cautiously for fibrosis screening in subjects older than 65 years. NITs are not the most accurate diagnostic tools for the identification of fibrosis. However, it must be kept in mind that elastography is a method not available in primary care, and there is a high level of agreement that the use of NITs, in particular FIB‐4, may be useful in excluding the risk of fibrosis. Additionally, in Italy, recent guidelines^30^ provide, as suggested by the European Association for the Study of the Liver,^27^ a two‐step referral pathway (NITs and elastography based on FIB‐4) to reduce the risk of overestimation with NITs.

## 5 CONCLUSION

Even if there is still an identification/registration gap for NAFLD in primary care, we found a high probability of fibrosis in NAFLD subjects in Italy. Owing to its burden, the increased awareness of NAFLD is an urgent need for primary care physicians because of the lack of standard disease codification methods, training and shared guidelines with specialists. Our data suggest that many cases with NAFLD have clinical comorbidities (cerebro‐cardiovascular and kidney diseases), which have a high impact on the quality of life and health system costs and may help explain the delay in referral for liver specialists. An interesting finding is a close association between cirrhosis and renal function deterioration. This should lead us to recommend extensive use of NITs also in order to identify early cases, including those with cirrhosis to be referred to hepatology centers. The deterioration of renal function and its association with NAFLD, widely reported in the literature, combined with the use of polypharmacy, should be a further element of awareness of the complexity of this type of patient and the need for a multidisciplinary approach, as recommended by the recent multi‐stakeholder position paper.^14^

Implementing simple NITs (FIB‐4 and NFS) in all the software used in primary care or automated laboratory reports may optimize the fast identification of patients at risk for liver and non‐liver‐related complications needing a specialistic consultation. Therefore, the role of GPs is crucial in correctly prescribing referrals to a liver specialist in this scenario.

## FUNDING INFORMATION

This work was supported by a project grant from Gilead Sciences (grant no. Gilead ISR IN‐IT‐989‐5338 to LM). Research activities of LM and AGa are supported by Ministero della Salute – Ricerca Corrente 2022, Fondazione Roma, intramural UCSC Linea D1, EU IMI2‐Horizon 2020 project LITMUS (grant agreement no. 777377).

## CONFLICT OF INTEREST

Luca Miele: Scientific advisor or consultant for Alfa‐Sigma, Boehringer‐Ingelheim, BMS, Echosens, Galmed, Gilead Sciences, IBSA, Intercept, MEDA, MyGenomics, MSD—Merck Sharp & Dohme, Novartis, Pfizer, Promethera, Rottapharm‐Madaus, Siemens Healthineers and Synageva; Francesco Lapi: Epidemiology consultant for Gilead and Abbvie. All other authors declare no competing interests.

## Supporting information


Table S1.
Table S2.
Table S3.
Table S4.

## Data Availability

Data sharing is not applicable.
