# Supplementary material for: Non‐alcoholic fatty liver disease and the risk of fibrosis in Italian primary care services: GPS‐NAFLD Study
Source: Liver Int. 2022 Oct 18;42(12):2632–45. doi: 10.1111/liv.15443 (PMC9827935; doi:10.1111/liv.15443)
Supplement: Supplementary file 1 — Table S1. Table S2. Table S3. Table S4. [file LIV-42-2632-s001.docx]

**SUPPLEMENTARY MATERIAL**

Supplementary Table 1. Algorithm for the selection of the study population (exclusion criteria)

| **Condition** | **ICD-9-CM diagnostic codes** | **Time of registration in relation to the index date** |
| --- | --- | --- |
| Alcohol abuse (>20 g/day) and/or alcohol-related diseases | 291*, 303*, 305.0, 357.5, 535.3, 571.0-3, 790.3, V11.3 | Preceding 2 years or in the following 6 months |
| Hepatitis B: | 070.2*, 070.3 |  |
| - HBV-DNA positive | – | Ever before or in the following 6 months |
| - HBs Ag positive | – | The year before or in the following 6 months |
| Hepatitis C: | 070.41, 070.44, 070.51, 070.54, 070.7* | – |
| - HCV-RNA positive | – | In the following 6 months |
| - Anti-HCV positive | – | Preceding 2 years or in the following 6 months |
| Autoimmune liver disease | 573.3; 571.4 coupled with the term “autoimmune” | – |
| Primary biliary cholangiopathy | 576.1 | – |
| Hepatic or extra-hepatic neoplasia | 140*,141*, 142*, 143*, 144*, 145*, 146*, 147*, 148*; 149*, 150*, 151*,152*, 153*, 154*,155*,156*,157*,158*,159*,160*,170*,171*,172*,173*, 174*,175*,176*,177*,178*,179*,180*,181*,182*, 183*,184*,185*,186*,187*,188*,189*,190*,191*, 192*,193*,194*,195*,196*,197*,198*,199*, 200*, 201*, 202*, 203*, 204*, 205*, 206*, 207*, 208*, 209*, 210*, 211*, 212*, 213*, 214*, 215*, 216*, 217*, 218*, 219*, 220*, 221*, 222*, 223*, 224*, 225*, 226*, 227*,228*, 229*, 230*, 231*, 232*, 233*, 234*, 235*, 236*, 237*, 238*, 239* | Before or in the 6 months following the index date |

## Supplementary Table 2. Comorbidities identified by ICD-9-CM either before or after the index date

| **Condition** | **I** **ICD-9-CM diagnostic codes** |
| --- | --- |
| Diabetes | 250* |
| Metabolic syndrome | 277.7 |
| Cerebro/cardiovascular disease | 433*, 434*, 436*, 438*, 342*, 410.0*, 410.1*, 410.2*, 410.3*, 410.4*, 410.5*, 410.6*, 410.7*, 410.8*, 410.9*, 411.0*, 411.1*, 429.7* |
| Heart failure | 428*, 402.01, 402.11, 402.91, 404.01, 404.91 |
| HIV diagnosis | 042, V08, 795.71 or HIV-positive test |

|  | **Female** | | | **Male** | | | **Total** | | |
| --- | --- | --- | --- | --- | --- | --- | --- | --- | --- |
|  | **Active population (n)** | **NAFLD cases (n)** | **Prevalence (%)** | **Active population (n)** | **NAFLD cases (n)** | **Prevalence (%)** | **Active population (n)** | **NAFLD cases (n)** | **Prevalence (%)** |
| **Total** | 44,427 | 3,949 | 8.89 | 25,679 | 3,122 | 12.16 | 70,106 | 7,071 | 10.09 |
| **Age (years)** | | | | | | | | | |
| 18–27 | 4,811 | 324 | 6.73 | 3,819 | 239 | 6.26 | 8,630 | 563 | 6.52 |
| 28–37 | 6,261 | 494 | 7.89 | 3,263 | 409 | 12.53 | 9,524 | 903 | 9.48 |
| 38–47 | 7,791 | 663 | 8.51 | 3,691 | 664 | 17.99 | 11,482 | 1,327 | 11.56 |
| 48–57 | 8,578 | 729 | 8.5 | 4,184 | 716 | 17.11 | 12,762 | 1,445 | 11.32 |
| 58–67 | 6,202 | 660 | 10.64 | 3,784 | 511 | 13.5 | 9,986 | 1,171 | 11.73 |
| 68–77 | 5,190 | 542 | 10.44 | 3,723 | 360 | 9.67 | 8,913 | 902 | 10.12 |
| 78–87 | 4,242 | 431 | 10.16 | 2,613 | 192 | 7.35 | 6,855 | 623 | 9.09 |
| ≥88 | 1,352 | 106 | 7.84 | 602 | 31 | 5.15 | 1,954 | 137 | 7.01 |

**Supplementary Table 3. Prevalence rate of NAFLD in Italian primary care patients in 2017 for subjects with BMI <25**

## Supplementary Table 4. Characteristics (at least age and gender) of the population enrolled in the study versus the population excluded due to lack of available data

|  | **HSI, n (%)** | **No-HSI, n (%)** |
| --- | --- | --- |
| Total: | 142,982 (100) | 1,337,353 (100) |
| - Males | 69,350 (48.5) | 628,597 (47.0) |
| - Females | 73,632 (51.5) | 708,756 (53.0) |
| Mean age (years) | 43.72 ± 15.89 | 39.9 ± 18.8 |
| Age (years): |  |  |
| - 18–27 | 26,908 (18.82) | 433,892 (32.44) |
| - 28–37 | 24,824 (17.36) | 264,369 (19.77) |
| - 38–47 | 30,585 (21.39) | 213,484 (15.96) |
| - 48–57 | 30,314 (21.2) | 156,956 (11.74) |
| - 58–67 | 20,628 (14.43) | 124,061 (9.28) |
| - 68–77 | 8,204 (5.74) | 91,667 (6.85) |
| - 78–87 | 1,432 (1) | 44,167 (3.3) |
| - ≥88 | 87 (0.06) | 8757 (0.65) |
